# Collective agency and positive political theory

**DOI:** 10.1177/09516298231203158

**Published:** 2023-09-20

**Authors:** Lars J. K. Moen

**Affiliations:** 27258 University of Vienna, Vienna, Austria

**Keywords:** Agenda control, group agency, judgement aggregation, social choice, strategic voting

## Abstract

Positive political theorists typically deny the possibility of collective agents by understanding aggregation problems to imply that groups are not rational decision-makers. This view contrasts with List and Pettit’s view that such problems actually imply the necessity of accounting for collective agents in explanations of group behaviour. In this paper, I explore these conflicting views and ask whether positive political theorists should alter their individualist analyses of groups like legislatures, political parties, and constituent assemblies. I show how we fail to appreciate the significance of strategic voting and agenda control by treating groups as agents. I, therefore, conclude that positive political theorists should cling to their individualist approach and maintain that groups are not agents.

## Introduction

Positive political theory provides formal models of collective decision-making based on a view of individuals as rational actors. Political outcomes are understood as results of individuals’ rational decision-making. The main objective is to construct models that predict how these individuals combine to produce collective outcomes (Amadae and Bueno de Mesquita, 1999: 270). Only individual agents, no group agents, are included in the models (Laver, 1997: 29). List and Pettit (2011), however, find this strictly individualist focus inadequate. To explain and predict collective decision-making, they argue, we must treat certain groups as agents that cannot be reduced to the individual agents constituting them. They therefore consider group agency an unignorable reality and conclude we should endorse ‘group-agent realism’.^1^

Interestingly, these opposing views are based on similar aggregation problems in social choice theory. Arrow's (1963) theorem implies that different majorities within the same group can contradict one another, and we therefore cannot ascribe a coherent will to the group based on aggregates of its members’ preferences. The group appears incapable of behaving as a rational agent. Riker (1982) rejects collective agency on these grounds and looks at the strategic behaviour of individuals in his analysis of collective decision-making. Shepsle (1992), similarly, stresses that Congress is a ‘they’, not an ‘it’. To understand how a legislature functions, we must look at the reasons legislators have for behaving as they do; we should not pretend the legislature itself has reasons. ‘Individuals have intentions and purpose and motives’, Shepsle (1992: 254) writes, ‘collections of individuals do not. To pretend otherwise is fanciful’.

For List and Pettit (2011), however, it is because a group cannot function on aggregates of individual judgements that we must treat it as an agent. Since the members’ judgements may not add up to a coherent collective will, a group-level procedure must be applied to ensure coherence. This procedure, in List and Pettit's view, works as a group mind reflecting on the input from the group members to produce a coherent set of judgements. These judgements cannot be attributed to the individual group members; they can only be attributed to the group itself. Groups in which individuals knowingly contribute to a collective decision procedure are therefore irreducible group agents. List and Pettit provide various examples of such groups, including groups much studied by positive political theorists, such as legislatures, political parties, and collegial courts. We cannot explain how these groups function merely by looking at their individual members, they argue. We must instead treat the groups as rational agents with their own beliefs and desires.

Writing before List and Pettit formulated their group-agent realism, Laver (1997: 29) states that rational choice theory's focus on analysing collective entities in terms of the individuals constituting them forces us to think harder about the metaphors, as he perceives them, of collective minds and agents. This reduction to the individual level, he says, is ‘[p]erhaps rational choice theory's single greatest virtue’. In this paper, I consider whether hard thinking about List and Pettit's group-agent realism will lead positive political theorists to soften their dismissive view of collective agency. Will a focus on certain groups as irreducible agents improve their understanding of group behaviour, as List and Pettit claim, or are they right to maintain their firm stance against collective agency? I argue for the latter view by showing how the social-choice results List and Pettit rely on do not, in fact, bring group minds into existence. Positive political theory's individualist view of groups provides important insight that is obscured from view if we follow List and Pettit in treating groups as agents.

Shepsle (1992) identifies four key questions for positive political theory emerging from social choice theory: ‘which majority wins’, ‘what role for procedures’, ‘who sets the agenda’, and ‘how to interpret revealed preferences’. I show how Shepsle and other rational choice theorists take the answers to the first two questions to support their rejection of collective agency. But the same answers lead List and Pettit to their group-agent realism. We shall see, however that answers to the last two questions undermine the case for group agency. If we treat collective-level procedures as autonomous group minds, as List and Pettit do, we shall fail to understand how individuals can use these procedures to their advantage. An agenda setter can effectively determine the outcome, and so can the preferences individuals reveal in their voting behaviour.

I therefore defend positive political theory's reductionist understanding of collective decision-making against the challenge from List and Pettit. Unless we reduce groups to individual agents and consider their rational behaviour within the group structure, we miss out on important insight into how groups function. Group agency is therefore an obstacle rather than an asset to the analysis of collective decision-making.

## Cycles and inconsistencies

Shepsle (1992) considers the decision-making in the U.S. Congress and rejects the idea of legislative intent. A legislator has intentions, he argues, but a group of legislators does not. We cannot explain why one bill rather than another was passed by looking at the legislature's intentions. The legislature, on this view, cannot be interpreted in terms of its intentional states (beliefs, desires, intentions, etc.) because it has none. One part of Shepsle's rejection of a Congressional group agent comes from Arrow's theorem: Any collective decision procedure satisfying conditions for fair representation of individuals’ transitive preferences can deliver an intransitive collective preference ordering. The group will then prefer some other alternative to every alternative. We therefore cannot say that the group prefers any alternative. There is a majority in favour of each alternative, and which majority is decisive is determined by the procedure, not by the group.

Pettit (2014) acknowledges that one account of group agency is undermined by Arrow's theorem, but this is merely a fictional, or metaphorical, account, he says. Hobbes (2008: ch. 16) thought a committee governed by a majority rule could function as a unified person representing the people. But as he also noted, this committee is a person merely ‘by fiction’, as it has no voice of its own. Its voice is really just that of the majority of the committee. The group is reducible to the individual persons constituting it and is therefore no actual person or agent. For Pettit, a demonstration of why a group cannot function as a rational agent based on majoritarian decision-making is to deny this fictionalist account of group agency.^2^

But List and Pettit see nothing fictional about the kind of group agency they defend. It is precisely because of the inconsistency of majority decisions that we should not focus on the group members but rather on the group itself as an agent. Their ontological view of group agents as real is based on the argument that the best social-scientific theories do not reduce these groups to individual agents but rather treat them as agents acting on their own beliefs and desires. Group agents are real because they are indispensable in our best attempts to explain social phenomena (List, 2021: 1215–1217). Such explanations, particularly concerning political institutions, is the focus of positive political theory.

List and Pettit focus especially on aggregation problems in their argument for group-agent realism, and particularly on a problem in judgement aggregation analogous to Arrow's theorem.^3^ Here individuals do not submit preference orderings but instead make binary judgements (true/false, yes/no, etc.) of propositions. The problem emerges when the propositions on the agenda are logically interconnected—that is, tied together by a logical connective, such as ¬ (not), ∧ (and), ∨ (or), → (implies), or ↔ (if and only if). For example, we connect the propositions *p* and *q* by adding a third, conjunctive proposition, ‘*p* ∧ *q*’, to the agenda. We assume that each individual holds consistent judgements of these propositions, so that no judgement precludes any other judgement. If the individual judges that *p* and *q*, for example, the individual will also judge that ‘*p* ∧ *q*’. But the majority judgements can nonetheless be inconsistent. The group might end up judging that *p* and *q*, but not ‘*p* ∧ *q*’, as we see in Table 1.

**Table 1.** Each group member submits a consistent set of judgements, but the majority judgements are nonetheless inconsistent.

	*p*	*Q*	*p* ∧ *q*
Member A	True	True	True
Member B	True	False	False
Member C	False	True	False
**Majority**	**True**	**True**	**False**

List and Pettit (2002) prove the general result that no decision procedure can ensure collective rationality without undermining the fair representation of the group members’ consistent sets of judgements. That is, no non-dictatorial procedure satisfying the three fairness conditions

**Universal domain:** The aggregation function admits as input any possible profile of individual attitudes towards the propositions on the agenda, assuming that individual attitudes are consistent and complete.

**Anonymity:** All individuals’ attitudes are given equal weight in determining the group attitudes. Formally, the aggregation function is invariant under permutations of any given profile of individual attitudes.

**Systematicity:** The group attitude on each proposition depends only on the individuals’ attitudes towards it, not on their attitudes towards other propositions, and the pattern of dependence between individual and collective attitudes is the same for all propositions.

will satisfy

**Collective rationality:** The aggregation function produces as output consistent and complete group attitudes towards the propositions on the agenda.^4^

It follows that the group cannot simply adopt its members’ judgements. To form complete and consistent judgements (collective rationality), the group's aggregation function must either restrict the set of acceptable logically possible profiles of individuals’ judgement sets (weaken universal domain), give more weight to some individuals’ judgements than to others’ (weaken anonymity), or the group's judgement of one proposition might depend on its judgement of another proposition, or propositions, on the agenda (weaken systematicity).^5^

Pettit (2001) calls this the discursive dilemma, as people coming together to make a collective decision are forced to choose between collective rationality and responsiveness to the group members’ judgements. The problem was first recognized by the nineteenth-century French mathematician Poisson (1837: 21), and later by Vacca (1921) and Kornhauser and Sager (1986). Elster (2015: 413–415) illustrates it with a case from the French constituent assembly of 1789–1791, where three sub-groups of roughly equal size were divided on the issues of whether to stabilize or destabilize the new regime, whether a unicameral or bicameral legislature would be more stabilizing, and ultimately whether to adopt a unicameral or bicameral system. Table 2 presents these issues as three interconnected propositions.

**Table 2.** Individuals in each of the three groups in the French constituent assembly hold consistent judgements but the majority judgements are nonetheless inconsistent.

	Bicameralism is stabilizing (*p*)	If bicameralism is stabilizing, then bicameralism (*p* → *q*)	Bicameralism (*q*)
Right-wing reactionaries	Yes	No	No
Centrist moderates	Yes	Yes	Yes
Left-wing radicals	No	Yes	No
**Majority**	**Yes**	**Yes**	**No**

We see that there is majority support for bicameralism being stabilizing and for the view that if bicameralism is stabilizing, then this system should be adopted. However, a majority rejects bicameralism and opts for unicameralism. We understand this inconsistency when we notice that the first two propositions are supported by different majorities and that the intersection of these majorities, containing only the moderates, is a minority. Only this minority therefore supports bicameralism. So, while each member holds a consistent set of judgements, the majority judgements are inconsistent.

The assembly did not actually vote on each of these issues. They instead voted directly on whether to adopt a unicameral or bicameral system, and unicameralism was therefore selected. We can see why the right-wing reactionaries and the left-wing radicals supported this decision, and we can also see why the centrist moderates supported bicameralism. But why did the assembly decide on a unicameral legislature? Since a majority wants the most stabilizing system, and a majority considers this to be bicameralism, the assembly's decision in favour of unicameralism appears irrational. The explanation for the decision, then, is simply that votes were taken only on the outcome. The decision would have been the opposite had it instead been based on the assembly members’ views of the ‘reasons’—that is, on whether stability is desirable and which system is most stable. No coherent set of collective beliefs and desires can therefore explain the decision that was in fact made. This is just as Shepsle, Riker, and other positive political theorists would expect.

But for List and Pettit, this is the wrong interpretation. There is no consistent set of majority-supported judgements on which to ground the collective decision, but that is no reason for denying that the assembly has reasons for its decisions. This observation just means the assembly's reasons are not reducible to those of its members. The reasons are there, but they are present only at the group level. This is why List and Pettit think we need to treat the group as an agent with its own reason for its decision. It cannot form its judgements directly from its members’ judgements. It must instead reflect on these judgements, as List and Pettit see it, and accept only some of them in order to ensure a coherent set of collective judgements. List and Pettit therefore understand the procedure to function as a group mind forming attitudes with a certain autonomy from the group members. We can then explain the group's decision on the outcome, *q*, by looking at its judgements of the premises, *p* and *p* → *q*, which it does not share with most of its members.

To make further sense of this view, let us consider procedures List and Pettit believe to function as a group mind. They opt for procedures violating systematicity (List and Pettit, 2011: 56–57). Systematicity combines neutrality and independence conditions familiar in social choice theory by requiring that the propositions on the agenda be treated independently and not in accordance with a certain pattern.^6^ A procedure that weakens this condition can therefore give priority to some proposition, or propositions. And different procedures can produce different outcomes based on the same profile of individuals’ judgements depending on the priority rule.

A conclusion-based procedure gives priority to the outcome, or conclusion, by accepting the majority judgement of the conclusion and then adopting the majority judgements of the premises only if they are consistent with the prioritized conclusion judgement. In the French constituent assembly, this procedure would result in bicameralism being rejected along with at least one of the other propositions. For List and Pettit, this means the group will have reasons for its decision, but its members will not.

If a premise-based procedure were applied, bicameralism would have been adopted since this procedure assigns to the group the judgement of the outcome that follows from the judgements of the premises. The constituent assembly would then form a desire for stability and judge that since it supports stability, it should adopt bicameralism, and therefore adopt bicameralism. Again, these judgements are said to belong to the group, not to its members.

We can see how the outcome depends on which procedure is employed. For List and Pettit, that means the group itself has a considerable degree of control over its own judgement formation. They see the procedure as a reflective group mind forming a set of judgements that cannot be ascribed to the group members. The particular procedure employed, then, is understood to generate judgements for the group as a distinct agent. The mistake Shepsle, Riker, and others seemingly make when they deny that a group can act on its own beliefs about relevant issues is that they try to understand the group's beliefs in terms of its members’ judgements. But by treating the group itself as an agent, the argument goes, the reasons for its decisions become apparent.

## Agenda setting

Social choice theory, as Shepsle (1992) remarks, leads us to ask not what *the* majority decides for the group, but *which* majority decides, and that is determined by the particular procedure employed. This procedure, in List and Pettit's view, operates as a group mind forming its own judgements, which we cannot understand simply as the judgements of most of the group members. The group, List and Pettit (2011: 76) say, thus possesses ‘an important sort of autonomy’. But this view becomes problematic when we now turn to the other two questions from social choice theory. Does it matter who sets the agenda and how? And how should we interpret the preferences individuals reveal in their voting? These questions concern the significance of how individuals behave within their group. When we consider agenda setting and, in the next section, strategic voting, it will become clear why collective decision-making is best understood at the individual level and why group agency is an obstruction in analyses of political decisions.

Agenda control and strategic voting are two aspects of manipulation. To control the agenda is to determine which alternatives or propositions appear on the agenda, how one is prioritized over another, or the order in which they are voted on. To vote strategically is to express a judgement one believes to be false in order to contribute to a more desirable outcome than what one would have contributed to by voting sincerely. Generally, an aggregation function is manipulable if and only if there is a logical possibility of a profile of individuals’ attitudes such that the outcome depends on how an individual sets the agenda (agenda control) or individuals can achieve a better outcome, from their own points of view, by voting untruthfully (strategic voting).

And manipulability is ubiquitous. Gibbard (1973) and Satterthwaite (1975) show that with three or more alternatives, any non-dictatorial decision procedure is manipulable. We can therefore understand why counterfactual reasoning in game-theoretic models is a central part of positive political theory, since manipulability gives actors an incentive to make choices with an eye on outcomes they want to achieve or avoid. Such reasoning is significant also in judgement aggregation. Dietrich and List (2007b) prove that with an agenda of interconnected propositions, an aggregation function that satisfies universal domain, collective rationality, responsiveness, and non-manipulability is a dictatorship of one individual.^7^ We must therefore expect individuals to behave strategically with the result in mind.

In this section, I discuss agenda control, and here I assume that individuals express their judgements sincerely. I also assume agenda setters have perfect information about other group members’ sincere judgements. There are then various ways in which the agenda setters can affect, or even determine, collective decisions. First, they can decide which propositions to feature on the agenda. In the constituent assembly example, a reactionary agenda setter can place just the outcome on the agenda, which was the agenda the assembly members actually were faced with. This is a conclusion-based procedure, where only the outcome is voted on. The reactionaries will thereby get the outcome they want. A moderate, however, can place just the first two propositions on the agenda by applying the premise-based procedure, which accepts majority judgements of the premises and then assigns to the group the judgement of the outcome that follows from these majority judgements. The outcome will then be the bicameral system, which the moderates favour. The important point here is that by treating the procedure by which the group makes its judgements as an autonomous group mind, we overlook the fact that the procedures are the strategic choice of some individuals.

With a certain domain restriction, however, such manipulation will be impossible. Black's (1948) median voter theorem shows that the problem of intransitivity that Arrow generalized does not occur when the profile of the individuals’ preferences is single-peaked. A preference profile is single-peaked if there is a way of ordering alternatives from left to right along a dimension so that for any individual, the lower an alternative is in the individual's preference ordering, the further away it is from the individual's ideal point. The collective preference ordering will then be transitive. List (2003) proves a similar result for judgement aggregation. With the structure condition ‘unidimensional alignment’, there is a way of ordering all individuals’ judgements of any proposition along a single dimension so that all positive judgements of a proposition, *p*, will appear either to the left or to the right of all negative judgements of *p*. With this domain restriction, List shows that proposition-wise majority voting will satisfy anonymity, systematicity, and collective rationality. The majority judgements will then be consistent, and an agenda setter cannot manipulate the outcome in the ways just described.^8^

In cases of the discursive dilemma, collective judgements are inconsistent and there is, therefore, no unidimensional alignment. In Table 3, we see one way of ordering the judgements of the factions of the French constituent assembly. The positive judgements of *p* are all to the right of the negative judgements of *p*, and all positive judgements of *p* → *q* are to the left of the negative judgement of *p* → *q*. But for *q*, a negative judgement appears on either side of the positive judgement. It is the inconsistency in the majority judgements, as we see in the far-right column, that List and Pettit rely on in their defence of group agency. But it is also what gives the agenda setter the power described above. We fail to appreciate this power of agenda control if we take the group itself to be an agent in control of its own judgement formation.

**Table 3.** The profile of the constituent assembly members’ judgements is not unidimensionally aligned.

	Left-wing radicals	Centrist moderates	Right-wing reactionaries	Majority
Bicameralism is stabilizing (*p*)	No	Yes	Yes	**Yes**
If bicameralism is stabilizing, then bicameralism (*p* → *q*)	Yes	Yes	No	**Yes**
Bicameralism (*q*)	No	Yes	No	**No**

However the significance of agenda control is not limited to the cases discussed above. We should also recognize that by selecting the propositions on the agenda, the agenda setter can effectively design a unidimensionally aligned or non-aligned profile of individuals’ judgements. Why is stability the evaluative criterion under consideration in the French constituent assembly? Assuming all propositions are on the agenda, is stability chosen because it benefits a moderate agenda setter? A reactionary or radical agenda setter might, for example, instead put efficiency on the agenda. Perhaps the reactionary thinks an efficient system could more swiftly reinstall the monarchy, and the radical might think it would enable more rapid implementation of a radical programme. The moderates might be sceptical about efficiency, but the radicals and reactionaries could form an unholy alliance to ensure consistent majority judgements for efficiency, for unicameralism being most efficient, and for unicameralism. Without altering anyone's judgements, changing the propositions on the agenda can thus turn non-alignment into alignment. Unicameralism will then be the decision under any procedure.

By following List and Pettit in treating the group as an agent in control of its own judgement formation, we fail to see the significance of the agenda setter's power. We might get the inconsistent majority judgements they believe to force a group mind into existence, but this could be the agenda setter's clever construction. Only at the individual level are we able to see how the decision was made. So, regardless of whether majority judgements are consistent or inconsistent, treating the group as an agent makes us lose sight of agenda setters’ influence on collective decisions.

## Strategic voting

Agenda control is important for understanding collective decisions, but when we weaken the sincere voting assumption, we realize that agenda-setting leaders leave other group members with significant scope for strategically shaping collective decisions. Leaders may control the agenda, but other members control their votes, and they can vote so as to enhance their influence on the outcome and reduce that of the agenda setter. Riker (1986: 149) therefore sees strategic voting as the ‘flip side’ of agenda control.

In this section, I show how individuals’ strategic voting can produce an outcome based on their preferences regardless of procedure and priority rule. Strategic voting has been much studied by positive political theorists, and List and Pettit (2011: ch. 5) also recognize that it might occur in the cases they base their group-agent realism on (see also Dietrich and List, 2007b; List, 2004: 505–510). They do not, however, consider how strategic voting problematizes their view of collective bodies as irreducible agents.

For List (2012: 209), ‘the French Constituent Assembly avoided an overt instance of inconsistent majority judgements by not taking explicit votes on all propositions; rather, it voted only on whether or not to introduce bicameralism’. This is correct only if we assume that the assembly members would have voted sincerely had all propositions been on the agenda. In light of the last section's discussion of agenda control, we would still not be right to see the assembly as a group agent. But if we for the moment set that matter aside, we can at least see why List and Pettit think we cannot make sense of the assembly's support for unicameralism by looking at its members’ sincere judgements, since they conflict with this decision.

But should we expect individuals to express the same judgements regardless of how many propositions there are on the agenda and of which procedure is employed? From the perspective of positive political theory, we take individuals to have a preferred outcome, and to vote so as to bring about this outcome. Given the facts about their social environment, they may see that misrepresenting their judgement of some proposition may help bring about their preferred outcome. This explains strategic voting and why judgements held and judgements expressed are conceptually distinct and do not always coincide. Assuming voters adopt equilibrium strategies, Austen-Smith and Banks (1991) show that any electoral rule is responsive to changes in their policy preferences, even if this is not so with respect to expressed preferences. We shall see below how outcomes are responsive to individuals’ preferences also in the procedures of judgement aggregation List and Pettit focus on.

Shepsle (1992) appreciates that individuals vote as they do with an eye on the outcome. He makes this point when he says legislators might, for example, support an amendment because they believe it would undermine the bill altogether, or perhaps because of a reasonable compromise between competing interests. There are therefore no grounds for suggesting there was a single collective legislative intent behind passing the bill.

Now, Shepsle takes this to mean we can never understand legislation to be based on legislators’ shared set of reasons. List and Pettit might then claim it is exactly because the legislators do not agree on reasons for their decision that we must ascribe reasons to the legislature and not to the legislators. But they do not mean to merely rationalize the legislature's decisions by ascribing supportive reasons to it, as that would be of little use for explaining how the decision was made. They instead, as we have seen, try to show that we explain the group's decision-making by looking at its judgements of propositions connected to its decision, and these judgements may not reflect the group members’ judgements.

If we assume legislation is made by such collective judgements of interconnected propositions, then Shepsle's case against legislative intent would be just as strong. The legislators would vote on the interconnected propositions, and we would not know why they voted as they did. They might have different reasons for endorsing or rejecting the same proposition. They might sincerely believe or like the proposition, or they might support it just because it would help them achieve what they believe to be the best possible outcome. The crucial observation is that by voting sincerely, individuals might contribute to an outcome they deem less desirable than the one they would contribute to by voting insincerely. And by realizing this, they might see an incentive to vote strategically. To best interpret a decision, then, we should not treat the group itself as an agent autonomously forming its own judgements. We should instead look at each member's actual and counterfactual voting behaviour to make sense of the collective judgements in terms of the preferences the individuals reveal by voting one way rather than another.

To illustrate this point, let us assume that each member of the French constituent assembly has perfect information about the procedure, the agenda, and the other members’ judgements of the propositions. We also assume that the members care more about some propositions on the agenda than about others. They have ‘outcome-oriented preferences’ if they care mostly about the outcome (bicameralism or unicameralism), and they have ‘reason-oriented preferences’ if they care mostly about the assembly's reasons for making its decision (Dietrich and List, 2007b). As I will now show, how individuals vote under a particular procedure will depend on their preference orientation. The outcome is therefore determined by their preferences and not by the procedure (Moen, 2023b). There is therefore no ‘group mind’.

With the premise-based procedure, no one with reason-oriented preferences has an incentive to vote strategically, as sincere voting can then only contribute to the collective judgements of the reasons they desire (Dietrich and List, 2007b: 290). But if the assembly members instead have outcome-oriented preferences, they will vote on the premises in whatever way they need to contribute to collective judgements coherent with their desired outcome (Ferejohn, 2007: 133–134). We can expect the reactionaries to strategically deny that bicameralism is stabilizing. They will then contribute to a majority view that bicameralism is not stabilizing, and the majority judgements, including the rejection of bicameralism, will be consistent. We can also expect the left-wing radicals to strategically deny the proposition ‘if bicameralism is stabilizing, then bicameralism’ to avoid contributing to an inconsistency that will result in their favoured outcome being rejected. As we see in Table 4, the result is consistent majority judgements in favour of the outcome the reactionaries and radicals want.

**Table 4.** Premise-based procedure and outcome-oriented preferences.

	Bicameralism is stabilizing (*p*)	If bicameralism is stabilizing, then bicameralism (*p* → *q*)	Bicameralism (*q*)
Right-wing reactionaries	Yes No	No	No
Centrist moderates	Yes	Yes	Yes
Left-wing radicals	No	Yes No	No
**Majority**	**Yes No**	**Yes No**	**No**

With the conclusion-based procedure, no one with outcome-oriented preferences has the incentive to vote sincerely, as the group will in any case adopt the majority judgement of the outcome. It might seem unlikely that the assembly members have reason-oriented preferences, but if they do, then the right-wing reactionaries and left-wing radicals have the incentive to misrepresent some of their judgements to avoid contributing to a result they do not want. If, for example, their proposition of concern is ‘bicameralism is stabilizing’, the reactionaries can accept the last two propositions, while the radicals can reject these two propositions (Table 5). And if their proposition of concern is ‘if bicameralism is stabilizing, then bicameralism’, then the reactionaries can strategically reject the first and last propositions, and the radicals can strategically accept these propositions (Table 6). There can, of course, also be cases where the reactionaries and radicals both have reason-oriented preferences but different propositions of concern. One of these subgroups will then vote as in Table 5, while the other will vote as in Table 6. In any case, we will get consistent majority judgements.^9^

**Table 5.** Conclusion-based procedure and reason-oriented preferences with *p* as proposition of concern.

	Bicameralism is stabilizing (*p*)	If bicameralism is stabilizing, then bicameralism (*p* → *q*)	Bicameralism (*q*)?
Right-wing reactionaries	Yes	No Yes	No Yes
Centrist moderates	Yes	Yes	Yes
Left-wing radicals	No	Yes No	No
**Majority**	**Yes**	**Yes**	**No Yes**

**Table 6.** Conclusion-based procedure and reason-oriented preferences with *p* → *q* as proposition of concern.

	Bicameralism is stabilizing (*p*)	If bicameralism is stabilizing, then bicameralism (*p* → *q*)	Bicameralism (*q*)?
Right-wing reactionaries	Yes No	No	No Yes
Centrist moderates	Yes	Yes	Yes
Left-wing radicals	No Yes	Yes	No Yes
**Majority**	**Yes**	**Yes**	**No Yes**

We see, then, that individuals vote so as to contribute to a set of collective judgements that are consistent with their judgement of their proposition of concern. The moderates never have an incentive to vote strategically. Their judgements are such that sincerity is their dominant strategy; regardless of the procedure and how others vote, sincerity can only contribute to their desired set of collective judgements. This is also evident in Table 3, where we see that the moderates’ judgements necessarily contribute to unidimensional alignment. And if the reactionaries and radicals vote so as to produce an inconsistency, we can understand them to reveal reason-oriented preferences under the premise-based procedure, or outcome-oriented preferences under the conclusion-based procedure.

The result will then depend on individuals’ preferences. With reason-oriented preferences, we will get the judgement set {*p*, *p* → *q*, *q*} either due to strategic voting in the premise-based procedure or due to the conclusion-based procedure's correction mechanism. With outcome-oriented preferences, the conclusion-based procedure selects unicameralism (¬*q*) and rejects at least one of the other two propositions: {¬(*p* ∧ (*p* → *q*)), ¬*q*}. And the premise-based procedure will deliver the same judgement set, since the radicals and reactionaries will ensure that a majority rejects one of the premises (Table 7).

**Table 7.** The same preference orientation will result in the same outcome regardless of procedure.

	Reason-oriented preferences	Outcome-oriented preferences
Premise-based procedure	*p*, *p* → *q*, *q*	¬(*p* ∧ (*p* → *q*)), ¬*q*
Conclusion-based procedure	*p*, *p* → *q*, *q*	¬(*p* ∧ (*p* → *q*)), ¬*q*

The question of how the group forms its judgements is, therefore, best answered by looking at individuals’ preferences, not by treating the procedure as a group mind working autonomously to ensure consistent collective judgements. We might get an inconsistency for the procedure to deal with, but we should then interpret that as a result of the group members’ preference orientation. We can get an inconsistency with the premise-based procedure if the group members have reason-oriented preferences, but not if they have outcome-oriented preferences. And the conclusion-based procedure can produce an inconsistency if the group members have outcome-oriented preferences, but not if they have reason-oriented preferences.

I do not mean to deny that it matters which procedure is employed or that one procedure can be considered superior to another. The focus on reasons in the reason-based procedure, especially when reasons are discussed in group deliberation, can enhance individuals’ understanding of an issue, and make them better able to make good decisions. This procedure has been found to be better suited than the conclusion-based procedure for making decisions based on sound and supportive reasons (Bovens and Rabinowicz, 2006; Pettit and Rabinowicz, 2001). Patty and Penn (2014) also defend reason-based collective decision-making. Despite inconsistent collective preferences, we can, on their view, reach a legitimate decision in a sequential procedure rejecting alternatives and ultimately selecting one based on an evaluative principle (Patty and Penn, 2014: chs. 5–6). Sound and supportive reasons can provide grounds for a legitimate outcome.

We can acknowledge these benefits of reason-based decision-making while interpreting outcomes in terms of individuals’ preferences. Better understanding of an issue may affect what outcome an individual prefers. Learning about others’ views in an open discussion of reasons may, for example, moderate, or ‘launder’, individuals’ preferences (Goodin, 1986: 87–89). It can result in more tolerance-based decisions widely acceptable in the society (Moen, forthcoming). But how preferences are formed does not alter the fact that decision-making is explainable in terms of individuals’ revealed preferences.

Now, List and Pettit (2011: ch. 5) are aware that individuals might lack an incentive to vote truthfully, and they acknowledge that the procedures they defend are manipulable since they violate systematicity. But they apparently do not consider this a problem for their group-agent realism. And since strategic voting leads to group judgements that may not reflect individuals’ sincere judgements, they might take it to support their position. Group members deliberating to figure out how to vote so as to avoid group-level inconsistency are, in List and Pettit's (2011: 61–64) view, parts of a group mind reflecting on its judgements to make them consistent. Perhaps they therefore understand individuals voting strategically to ensure collective judgements consistent with their propositions of concern as parts of a group mind.

But we cannot explain the outcome as the result of a group mind's reflection. The procedure may not be responsive to the group members’ sincere judgements, but it is, as we have seen, responsive to the group members’ preferences. Attributing reasons to a group agent can rationalize the outcome post hoc, but to explain it, we must look at individuals’ preferences about the outcome that drive their motivation for voting strategically. We must consider how they might deliberately misrepresent a judgement to contribute to the result they prefer. They control the collective decision-making on the basis of their preferences and allow the group itself no autonomy.

## Conclusion

List and Pettit ground their view of political groups as agents on a kind of aggregation problem that positive political theorists have traditionally understood to undermine any case for collective agency. Individuals vote for or against a set of propositions connected as reasons for a conclusion, or outcome. Each individual's judgements are consistent, but the majority judgements might nonetheless be inconsistent. Majorities in the French constituent assembly wanted the most stable system, judged this to be bicameralism, but then decided on unicameralism. Riker, Shepsle, and other positive political theorists would presumably say that the assembly has no reasons for its decision; only the assembly members have reasons for how they vote. But for List and Pettit, this is the wrong interpretation. Majority judgements of the premises conflicting with the majority judgement of the outcome means the assembly members do not collectively hold reasons for their support for unicameralism. To explain how the decision is made, we must therefore, on List and Pettit's view, ascribe reasons to the assembly that cannot be attributed to its members. This is to treat the assembly as a group agent.

In this paper, I have argued that positive political theorists should reject this account of group agency by showing how it collapses when we consider the significance of agenda control and strategic voting. By treating the group as an agent capable of forming its own mental states, we overlook how group members act strategically to help bring about collective outcomes in accordance with their preferences. By accounting for individuals’ strategic behaviour, we see that while we might get the kind of inconsistency List and Pettit believe gives rise to a group agent, this is because of how the agenda setter has set the agenda or because of how group members vote based on their preferences. We therefore cannot say a group agent controls the collective judgement formation. A group makes its decisions based on the various reasons its members have for supporting one outcome rather than another. We miss this important observation by treating the group itself as a reasoning agent. We should therefore maintain that a legislature, a constituent assembly, or any other group studied by positive political theorists is a ‘they’, not an ‘it’.

## Notes

1. While there is a dispute over the status of group agents, there is no significant disagreement here regarding what an agent is. I shall proceed on the assumption that both parties conform to the common view of agency List and Pettit (2011: 20) describe. On this view, an agent is a system or entity with representational states, or beliefs, about its environment, motivational states, or goals or desires, about how it wants its environment to be like, as well as a capacity to shape its environment in order to achieve its goals or satisfy its desires. Insofar as a group is an agent, then, it has desires it acts to satisfy on the basis of its beliefs about its environment.

2. I explore this group-agent fictionalism further in Moen (2023a).

3. For a brief survey of the literature on judgment aggregation, see Pigozzi (2015).

4. The conditions as stated here are quoted from List and Pettit (2011: 49).

5. This result differs from Arrow's theorem by excluding the Pareto principle and by requiring additional neutrality and anonymity conditions (Dietrich and List, 2007a). The systematicity condition combines neutrality and independence of irrelevant alternatives, or propositions. Informally, independence of irrelevant propositions requires that each proposition on the agenda be treated independently without regard for judgments of other propositions, while neutrality requires that changing the order in which the propositions appear on the agenda will not affect the result.

6. I define neutrality and independence in note 5 above.

7. Responsiveness here requires that for any admissible profile, if one or more voters change their votes in some direction, while other votes remain unchanged, the collective decision cannot change in the opposite direction. Weakening this condition would imply a degenerate aggregation rule (Dietrich and List, 2007b: 283).

8. Unidimensionality does not, however, imply that strategic voting cannot occur. Penn et al. (2011) show for preference aggregation that single-peakedness does not entail that voters will not vote strategically.

9. Here we also see that the result does not depend on the reactionaries and radicals having the same preference orientation. When group members vote strategically, they will vote consistently for or consistently against propositions on the agenda. Some members are then bound to combine to ensure a consistent set of majority attitudes. In the French constituent assembly, the radicals and the reactionaries will combine with each other, or at least one of them will combine with the centrists, to ensure a consistency result.
